# Development of a Spatial Decision Support System for Real-Time Flood Early Warning in the Vu Gia-Thu Bon River Basin, Quang Nam Province, Vietnam

**DOI:** 10.3390/s20061667

**Published:** 2020-03-17

**Authors:** Hong T. Nguyen, Trung Q. Duong, Liem D. Nguyen, Tram Q. N. Vo, Nhat T. Tran, Phuong D. N. Dang, Long D. Nguyen, Cuong K. Dang, Loi K. Nguyen

**Affiliations:** 1 Hanoi University of Science, Vietnam National University, Hanoi City 11416, Vietnam; nthong@vnu.edu.vn; 2 School of Electronics, Electrical Engineering and Computer Science, Queen’s University Belfast, Belfast BT7 1NN, Northern Ireland, UK; trung.q.duong@qub.ac.uk; 3 Faculty of Environment and Natural Resources, Nong Lam University, Ho Chi Minh City 71308, Vietnam; nguyenduyliem@hcmuaf.edu.vn; 4 Research Center for Climate Change, Nong Lam University, Ho Chi Minh City 71308, Vietnam; 5 Faculty of Information System and Remote Sensing, Ho Chi Minh City University of Natural Resources and Environment, Ho Chi Minh City 72107, Vietnam; ttnhat@hcmunre.edu.vn; 6 Institute of Research and Development, Duy Tan University, Da Nang City 50356, Vietnam; nguyendinhlong1@duytan.edu.vn; 7 Faculty of Information Technology, Nong Lam University, Ho Chi Minh City 71308, Vietnam; dkcuong@hcmuaf.edu.vn

**Keywords:** automated flood warning system, decision support system, flood forecasting, HEC–RAS, SWAT

## Abstract

Vu Gia-Thu Bon (VGTB) river basin is an area where flash flood and heavy flood events occur frequently, negatively impacting the local community and socio-economic development of Quang Nam Province. In recent years, structural and non–structural solutions have been implemented to mitigate damages due to floods. However, under the impact of climate change, natural disasters continue to happen unpredictably day by day. It is, therefore, necessary to develop a spatial decision support system for real-time flood warnings in the VGTB river basin, which will support in ensuring the area’s socio-economic development. The main purpose of this study is to develop an online flood warning system in real-time based on Internet-of-Things (IoT) technologies, GIS, telecommunications, and modeling (Soil and Water Assessment Tool (SWAT) and Hydrologic Engineering Center’s River Analysis System (HEC–RAS)) in order to support the local community in the vulnerable downstream areas in the event of heavy rainfall upstream. The structure of the designed system consists of these following components: (1) real-time hydro-meteorological monitoring network, (2) IoT communication infrastructure (Global System for Mobile Communications (GSM), General Packet Radio Service (GPRS), wireless networks), (3) database management system (bio-physical, socio-economic, hydro-meteorological, and inundation), (4) simulating and predicting model (SWAT, HEC–RAS), (5) automated simulating and predicting module, (6) flood warning module via short message service (SMS), (7) WebGIS, application for providing and managing hydro-meteorological and inundation data, and (8) users (citizens and government officers). The entire operating processes of the flood warning system (i.e., hydro-meteorological data collecting, transferring, updating, processing, running SWAT and HEC–RAS, visualizing) are automated. A complete flood warning system for the VGTB river basin has been developed as an outcome of this study, which enables the prediction of flood events 5 h in advance and with high accuracy of 80%.

## 1. Introduction

Among the countries in the world most affected by extreme weather events in the period from 1996 to 2015, Vietnam is ranked eighth [1]. In Vietnam, flooding is one of the major threats that have a negative impact on the development of the country [2]. In recent years, natural disasters including floods have increased in both intensity and variety due to the changes in global climate and socio-economic development activities [3,4,5,6]. The VGTB river basin is located in the mid-central region of Vietnam, and is the key economic zone of the region. The geographical location provides advantageous conditions for the socio-economic development of the province. However, the basin is seriously affected by natural disasters, which have had negative impacts on the economic growth rate of Quang Nam Province. According to five-year statistics from 2003 to 2007 by the Quang Nam Steering Committee for Storm and Flood Prevention and Control [7], the losses due to natural disasters in Quang Nam Province were estimated up to 6.26% of the province’s GDP. These consequences suggest that systematic studies are needed to find out the causes that lead to flooding and factors that reduce losses. Although there have been various studies on flood risks, there are still limitations in intensive multi-disciplinary research in this area. Specifically, while recording, calculating, and evaluating extreme events of flooding in 1999, 2007, 2009, and drought in 2005 could help find out the factors that caused those events, there were no methods of forecasting the events in real-time or alerting stakeholders in the relevant impacted areas. This is even more crucial in the context of global climate change, a result of which is serious variations in the flow of the rivers locating in the study area. Flood-related losses may reach higher due to the frequency and severity associated with climate change, making building a flood warning system for the VGTB river basin especially important.

With respect to hydrologic models, distributed hydrologic models such as Soil and Water Assessment Tool (SWAT) have more advantages over lumped hydrologic models thanks to their ability to capture the diverse characteristics of river basins and hydro-meteorological forcings, and as a result, are widely used to illustrate the physical mechanisms of floods in reality [8]. In flood warning systems, hydrologic and hydraulic models using data on the updated state of a watershed and the forecast of input data (precipitation and other parameters) are essential to the calculation of flood flow and the depth on the whole basin [9]. However, distributed hydrologic models do not take into account the physical characteristics of the rivers or channels, and instead, rely on a conceptual channel system for water routing in flood modeling [4]. To overcome this drawback, hydraulic models such as Hydrologic Engineering Center’s River Analysis System (HEC–RAS) have been added together with SWAT. The aim is to simulate the flows based on the topography of the channel and floodplain [4].

SWAT and HEC–RAS models have been extensively adopted in real-time flood modeling and forecasting. Rainfall distribution algorithms were proposed to project precipitation using data from auto weather stations and rainfall/runoff hydrological models in SWAT [10]. The HEC–RAS model was applied to design a near real-time flood monitoring system for the Marikina river basin, Philippines [11], or incorporated with Shuffled Complex Evolution to represent a real-time updating procedure for streamflow forecasting in the Lower Iguazu basin, Brazil [12]. For customizing HEC–RAS, Leon and Goodell [13] developed scripts in MATLAB to automate writing and reading input/output files, make plots, implementing parallel computations of the processes in their model. A real-time flood forecasting and flood inundation mapping for the Bagmati river system of Bihar, India was designed that integrated the SWAT and HEC–RAS models [14].

In a parallel research stream, flooding in the VGTB has received considerate attention via the applications of various flood simulation models, for instance, using SWAT in flood control with an emphasis on the role of forests; applying HEC–HMS and HEC–RAS models in flood simulation at VGTB [15,16,17]; using MIKE11 and HEC–RAS in flood modeling in the A Vuong river [18] or the Bung 4 river [19]; incorporating WetSpa and HEC–RAS to forecast flooding events in real-time [20]; integrating MIKE NAM, MIKE 11, and HEC–ResSim models into weather forecast model in order to predict flooding events [8]. However, until now, there has been no research that considers both SWAT and HEC–RAS in real-time flood simulation and prediction in this basin.

Hence, our research attempts to address this need at the VGTB river basin by designing a flood warning system through the Geographic Information System–Information Technology (GIS–IT) and integrating the SWAT and HEC–RAS model approach. The deployment of a flood warning system is very urgent to ensure the social-economic development sustainability of the VGTB river basin in the context of climate change. The objectives of this study are to determine vulnerability flood areas and peak flooding in the VGTB river basin and support farmers in vulnerable flood areas.

## 2. Study Area

**Figure 1:**
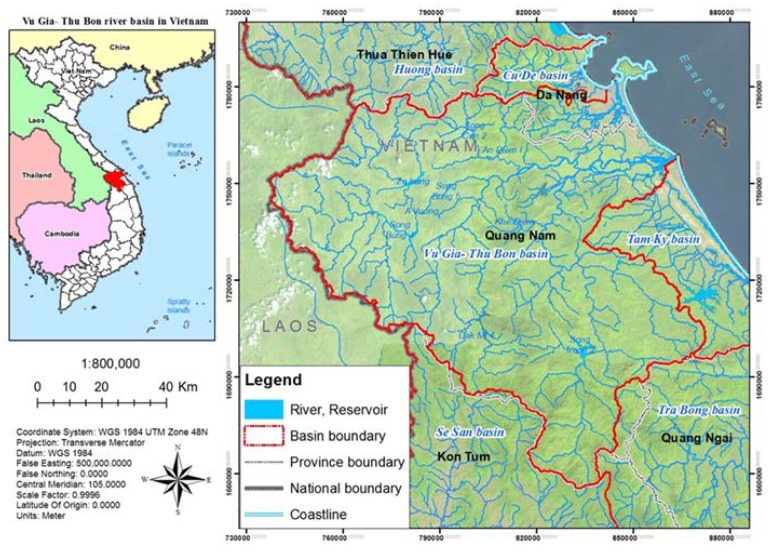
Geographic location of the Vu Gia-Thu Bon (VGTB) river basin [21] (reproduced with permission from Nguyen Kim Loi, Nguyen Duy Liem, Le Hoang Tu, Nguyen Thi Hong, Cao Duy Truong, Vo Ngoc Quynh Tram, Tran Thong Nhat, Tran Ngoc Anh, and Jaehak Jeong, Journal of Water and Climate Change; published by IWA Publishing, 2019).

The study area is the VGTB watershed, which is located in Central Vietnam. This watershed, with an area of roughly 10,000 km^2^, lies approximately between 14°95′ and 16°06′N in latitude and 107°21′ and 108°46′E in longitude, encompassing Da Nang City, Quang Nam Province, Quang Ngai Province, and part of Kon Tum Province (Figure 1) [8]. Based on the preliminary analysis of long-term temporal variations of rainfall and streamflow, it is noticeable that the annual flood occurrences normally last from October to December. This hazardous event poses a major threat to human lives and assets. A critical concern added to this landscape is the hydropower development in the watershed [7]. The main factors which contribute to flooding in the VGTB river basin include: (1) mountainous terrain is predominant, resulting in a dense, short, steep river system, and rapid flooding; (2) the coastal plain is narrow, the river mouth changes seasonally, leading to slow flood drainage; (3) effects of many forms of weather (typhoons, tropical low pressure, cold air, Northeast monsoon) cause heavy and long rains which are concentrated in the rainy season; (4) land-use activities (riverine infrastructure construction, primary forest clearance, hydropower dam development, mineral exploitation) increase the incidence and severity of floods.

## 3. Methodology

**Figure 2:**
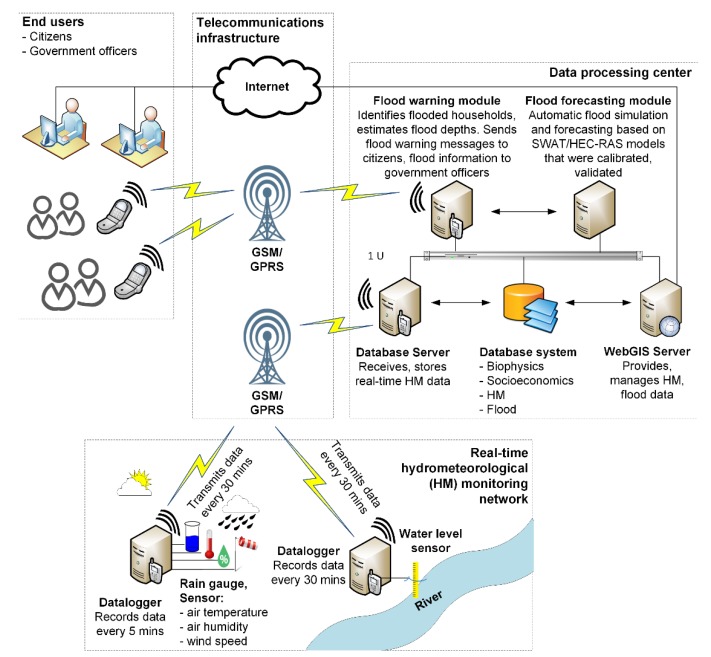
Flood warning system in the Vu Gia-Thu Bon river basin.

This study presents a spatial decision support system (SDSS) for real-time flood warnings in the VGTB river basin. We built upon our previous work [21,22] to design a system that incorporates both a hydrologic (SWAT) and a hydraulic (HEC–RAS) model. We developed a set of control scripts, and further, a user USACE HEC–RAS Controller [23] and Mouse Tracking. The following procedure is employed in this study: (i) create input files for SWAT model and then implement; (ii) use output files of SWAT model to create input files for HEC–RAS model, and implement HEC–RAS model; and (iii) visualize the floodplain map online. The framework in SDSS for real-time flood warning is shown in Figure 2 with 3 components: real-time hydro-meteorological (HM) data monitoring network, data processing center, and telecommunication infrastructure. In line with our previous research [21,22], the number of additional rain gauges should be set as 20 and the optimum number of hydrological stations for the VGTB river basin should be set as 5.

The flood warning system in the VGTB river basin includes 3 components: real-time hydro-meteorological (HM) data monitoring network, data processing center, and telecommunication infrastructure. The real-time hydro-meteorological data monitoring network includes a data logger for recording data every 5 min, rain gauges, and sensors (comprised of rainfall, air temperature, air humidity, wind speed sensors) from 20 meteorological stations, and water level data recorded every 5 min from 5 hydrological stations. All data is transmitted every 30 min to the data center by Global System for Mobile Communications (GSM)/ General Packet Radio Service (GPRS). The data processing center includes a data server and will receive and store data from stations. This is also where the automatic flood simulation and forecast based on SWAT and HEC–RAS models were calibrated and validated.

### 3.1. Meteorological Network

In practice, there are 9 stations at the VGTB river basin including in Ai Nghia, Hien, Hiep Duc, Hoi Khach, Kham Duc, Nong Son, Tien Phuoc, Thanh My, and Tra My. The statistic record of annual rainfall (1980-2013) has pointed out that there are several inaccuracies in the previous works with an error of about 7.47%. To lower this error rate to an acceptable level of 5%, data from at least 20 additional rain gauges [22] were required. The aim of the optimum rain-gauge network is to obtain all quantitative data averages and extremes that define the statistical distribution of the meteo-hydrological elements, with sufficient accuracy for practical purposes. The number of additional rain-gauge stations should be distributed in the different zones (caused by isohyets) in proportion to their areas, i.e., depending upon the spatial distribution of the existing rain-gauge stations and the variability of the rainfall over the basin [22].

**Figure 3:**
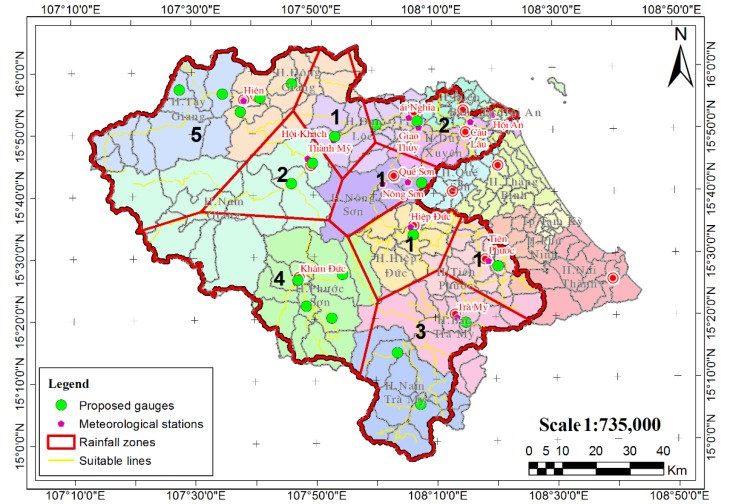
Meteorological stations network on the Vu Gia-Thu Bon river basin [22] (reproduced with permission from Nguyen Thi Hong, Phan Thi Thanh Truc, Nguyen Duy Liem, and Nguyen Kim Loi, International Journal on Advanced Science Engineering Information Technology; published by Indonesian Society for Knowledge and Human Development, 2016).

More importantly, our analysis using Thiessen’s interpolation method has also shown that the VGTB river basin has 9 different rainfall zones. Thus, based on the weight of rainfall zone areas, the number of stations should be different for each rainfall zone. Specifically, the additional numbers of stations for nine zones of large rainfall areas in the VGTB river basin were set as 1, 5, 1, 1, 3, 2, 4, 1, and 2 for Ai Nghia, Hien, Hiep Duc, Hoi Khach, Kham Duc, Nong Son, Tien Phuoc, Thanh My, and Tra My, respectively. Next, embedded maps on GIS environments such as hydrography, wind directions, transportation, and sub-basin were exploited to determine appropriate locations for the required stations. As a result, the optimum numbers and locations of meteorological stations have been achieved in our study, with suitable places to set up our station for each range of rainfall areas, as shown in Figure 3 [22].

### 3.2. Hydrological Network

**Figure 4:**
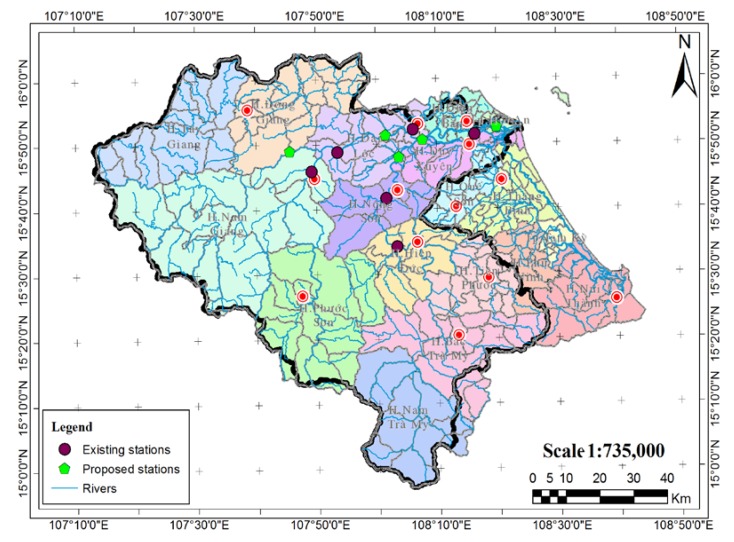
Location of hydrological stations on the Vu Gia-Thu Bon river basin [22] (reproduced with permission from Nguyen Thi Hong, Phan Thi Thanh Truc, Nguyen Duy Liem, and Nguyen Kim Loi, International Journal on Advanced Science Engineering Information Technology; published by Indonesian Society for Knowledge and Human Development, 2016).

From the previous analysis, the optimal number of stations was set as 5 based on the hydrological station criteria as in the research of [22]. These criteria were based on the stable operation ability of the station in deployment on a branch of the main channel and a section of channels in the basin. Similar to the meteorological network, five hydrological stations were set up following our field trip to two branches of channels, namely Vu Gia river and Thu Bon river, as shown in Figure 4 [22].

### 3.3. SWAT Model

The SWAT model is a semi-distributed model with the purpose of simulating the hydrology, sediment, and nutrient yield in large agricultural catchments with various agro-climatic conditions [24]. In this model, the hydrological processes are simulated at each hydrological response unit (HRU) using the water balance equation with hydrological components, consisting of precipitation, evapotranspiration, infiltration, groundwater, and surface runoff. A more elaborate explanation of the hydrological processes is given in the SWAT Theoretical Documentation [24].

#### 3.3.1. Overview of SWAT Input/Output Files

Input/output files of the SWAT model are organized as follows. Input files are classified into two categories: configuration files (file.cio, *.fig) and data input files (*.bsn, *.pcp, etc.). Concerning flood flow simulation, the relevant input files include master River Basin file (file.cio), and weather input files (*.pcp, *.tmp, *.slr, *.wnd). After each SWAT simulation, the input.std file, the output.std file, the output.hru file, the output.sub file, and the output.rch file are generated in the output. Meanwhile, the print codes of the master River Basin file (file.cio) manage the detail of the data printed out in the above files. Thus, the output files may aggregate daily, monthly, yearly, or for the entire simulation period based on the selected print code. For flood flow simulation, the reach output files are considered.

#### 3.3.2. Overview of HEC–RAS Input/Output Files

The input/output files of the HEC–RAS model are organized as follows: For unsteady flow simulation, the input files include geometry (*.g01), unsteady flow (boundary and initial conditions) (*.u01), and plan files (*.p01). The output files include water surface profiles, storage–outflow information, computed rating curves, etc., in a separate binary format (HEC and HDF5) or DSS format.

#### 3.3.3. Automated Procedure for SWAT Model

**Figure 5:**
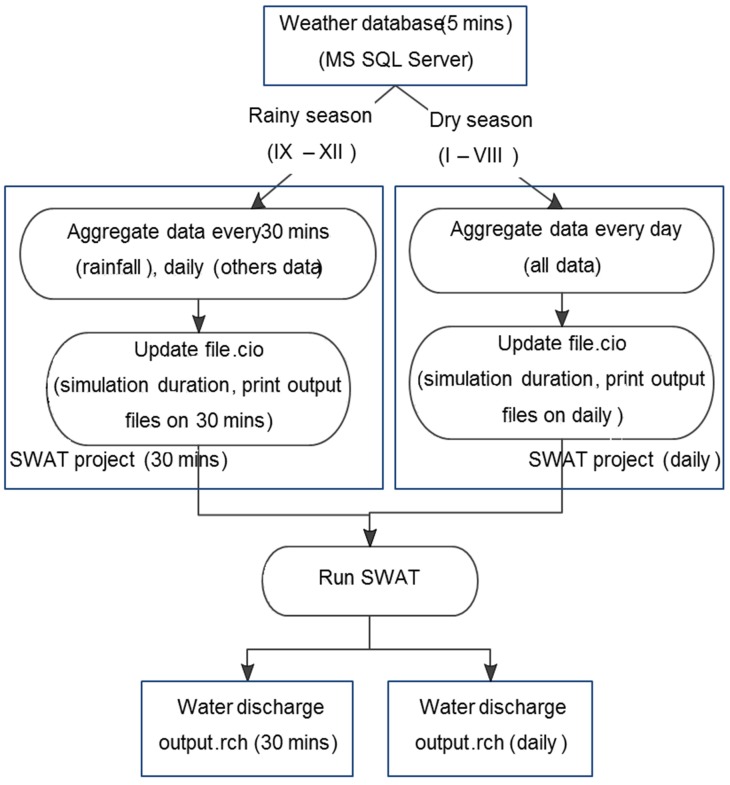
Automated process of streamflow simulation in the Soil and Water Assessment Tool (SWAT) model.

The automated procedure for streamflow simulation in SWAT consists of three steps, differentiated by season (rainy or wet season, see Figure 5): (1) real-time weather data (recorded every 5 min) and stored at the MS SQL Server database management system is aggregated into weather input files, in accordance with a predetermined interval of 30 min (for rainy season) or daily (for dry season); (2) simulation duration is updated such that the last date of weather data updates and the end day of simulation should match; then, the frequency of output files is printed in the master River Basin file every 30 min or daily; and (3) the SWAT model is run to simulate streamflow stored in the main channel output file.

#### 3.3.4. Writing SWAT Input Files

SWAT model relies on a vast amount of data as input. For flood warnings, due to its dynamically changing nature, weather data for SWAT should be updated in real-time, whilst other data of slow change nature such as topography, land-use, or soil may be periodically (yearly) monitored. Once the first SWAT scenario is completed, the entire input and output files will be stored in the TxtInOut folder in ASCII format. Users can easily interact with them through the use of simple scripts of reading or writing input files.

The SWAT model requires a large amount of input data such as topography, land-use, soil, and weather in order to run. For flood warnings, weather data needs to be monitored in real-time due to its continuous change. Meanwhile, the other data can be updated periodically (yearly) due to its slow change. After completing the first SWAT scenario, the entire input and output files are stored in the TxtInOut folder in ASCII format. The user can easily interact with them by using simple scripts of reading or writing input files. Input files relevant to this study include the master configuration file and weather data input files. The rainfall data at each station is stored for a given time: 30 min, or daily. To run SWAT, the script System.Diagnostics.Process.Start () is used for active SWAT executable. The reach output file is one of the output files generated in every SWAT simulation. By using simple scripts of reading input files in the library System.IO.Files, the user can extract streamflow data from the reach output file to write HEC–RAS input files.

#### 3.3.5. Automated Procedure for the HEC–RAS Model

**Figure 6:**
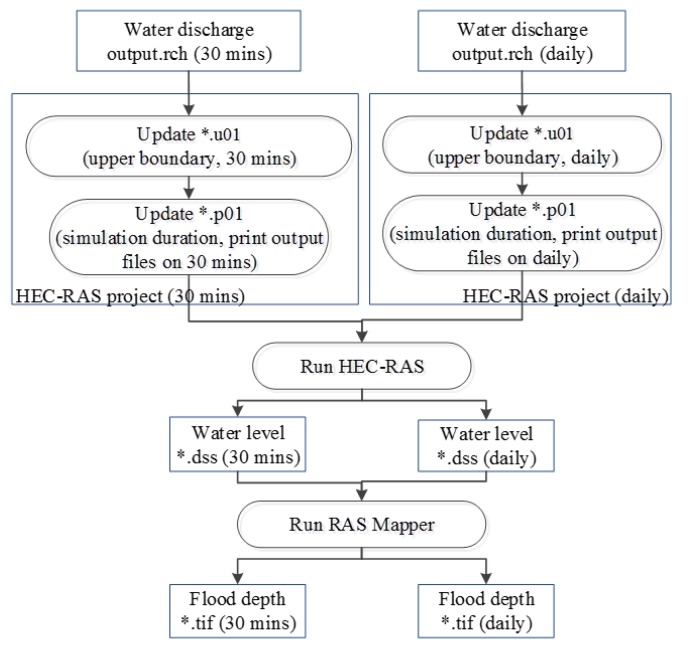
Automated process of water level, flood depth simulation in the Hydrologic Engineering Center’s River Analysis System (HEC–RAS) model.

Figure 6 illustrates the automated procedure that was used in the simulation of water level and flood depth (in the rainy or dry season) based on the HEC–RAS model, in four steps as follows: (1) by using water discharge data extracted from the SWAT model’s main channel output file, the boundary and initial conditions of cross sections in the unsteady flow data will be updated; (2) simulation duration will be updated such that the end day of simulation and the last date water discharge data has been updated should match, then the frequency of output files will be printed plan file; these will be implemented every 30 min (rainy season) or daily (dry season); (3) HEC–RAS model is run to simulate water level stored in DSS format; (4) RAS Mapper will be run to map flood depth stored in GeoTiff format. The file name matches the inundation time.

#### 3.3.6. Writing HEC–RAS Input Files

The input files of the HEC–RAS model are also stored in ASCII format. Users may interact with the input files by using simple scripts of reading or writing input files. For flood warning, the related input files are plan file (*.p01), and unsteady flow (boundary and initial conditions) (*.u01).

#### 3.3.7. Executing the HEC–RAS Model

Unlike SWAT, HEC–RAS runs on Windows Forms, which is different from Console scripts. As a result, users are required to interact with HEC–RAS on its interface, making the automation of active HEC–RAS process quite complex. To execute HEC–RAS automatically, this study uses the HEC–RAS Controller Code module that controls HEC–RAS through the macros written in VBA and embedded in file HECRASControllerCode_h2ls.xlsm. After the input data of HEC–RAS is created, the library Microsoft.Office.Interop.Excel is used to read file HECRASControllerCode_h2ls.xlsm and activate a macro Compute_CurrentPlan in order to run the HEC–RAS model.

Once the calculation of water level at cross-sections is finished, the Mouse Tracking technique is used to collect the users’ mouse cursor positions on the computer when they perform inundation mapping in the RAS Mapper tool manually. This is to automatically estimate flood depth in this application.

#### 3.3.8. Visualizing the Online Floodplain Map

The results from running RAS Mapper application is shown flood depth in GeoTiff format. This file is then uploaded to WebGIS by using the GeoTIFF module of GeoServer.

## 4. Results and Discussion

### 4.1. Automated SWAT and HEC–RAS Module for Real-Time Flood Forecasting

**Figure 7:**
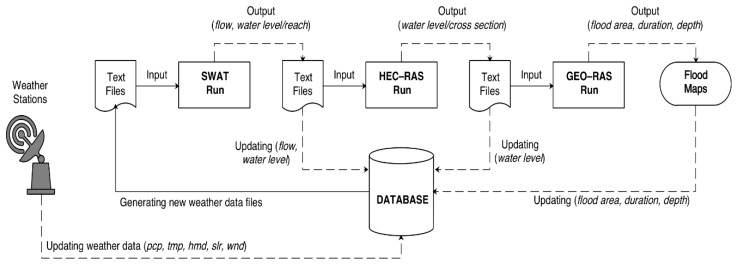
SWAT HEC–RAS module for real-time flood forecasting.

**Figure 8:**
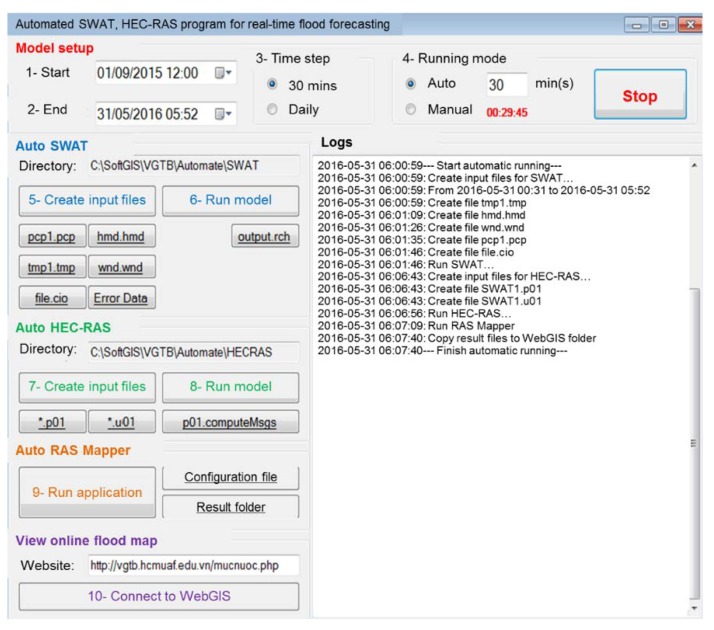
Automated SWAT and HEC–RAS module for real-time flood forecasting.

The general SWAT HEC–RAS module for real-time flood forecasting is shown in Figure 7. To facilitate system testing and troubleshooting, individual modules are packaged into a single one, as shown in Figure 8, with two running modes (i.e., manual and automatic). In the former mode, users perform all the tasks manually, while in the latter, no human intervention is required.

The application’s interface is organized as follows: Firstly, the model is set up by declaring start time, end time, time step (30 min/daily), and running mode (automatic/manual). By default, the start time is date 2015/09/01 (time when automatic meteorological stations transmit data fully, validly), end time is assigned to the system’s current time. Secondly, the start/stop is turned on and automatic running mode is turned on/off. Thirdly, auto SWAT is activated by creating input files, running model, linking to input files (pcp1.pcp, tmp1.tmp, hmd.hmd, wnd.wnd, file.cio), errors report of input data (Error Data), and link to output file (output.rch). Then input files, run model, link to input files (*.p01, *.u05), and results report of running model (p01.computeMsgs) are created using the auto HEC–RAS. Next, we use the auto RAS Mapper to run the application, link to configuration file “Mouse Tracking”, result folder. After that, it is necessary to view the online flood map by using “Connect to WebGIS” function to view the flood depth map. Lastly, logs display timeline for step-by-step implementation and start/finish status of the whole process.

### 4.2. Automated Floodplain Mapping in the Flood Season of 2015

**Figure 9:**
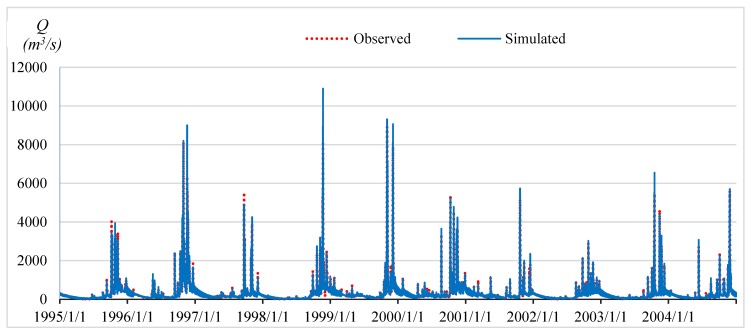
The calibration water discharge at the Nong Son hydrological station during 1995–2004.

**Figure 10:**
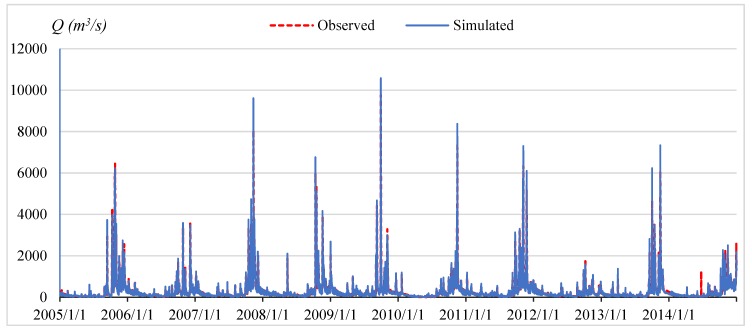
The validation water discharge at the Nong Son hydrological station during 2005–2014.

**Figure 11:**
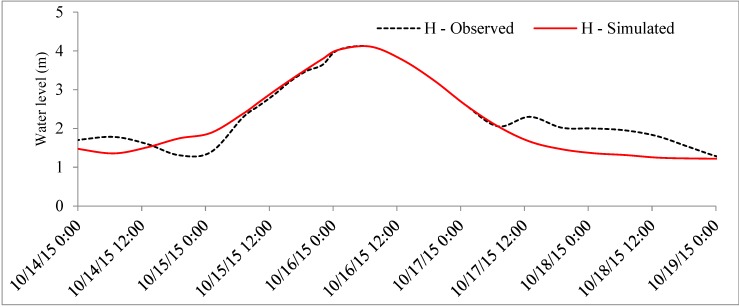
The calibration water level at the Cau Lau hydrological station at the VGTB river basin during October 14–19, 2015 flood event.

**Figure 12:**
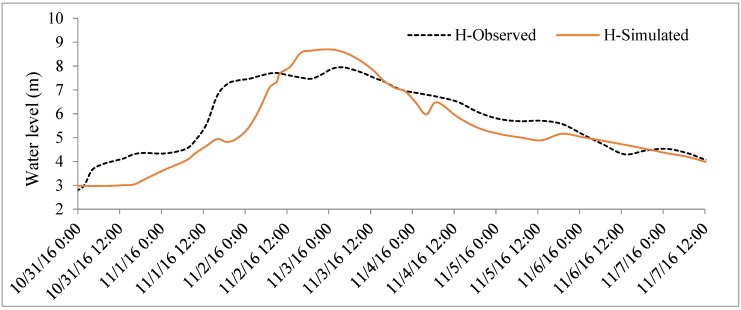
The validation water level at the Cau Lau hydrological station in the VGTB river basin during October 31–November 7, 2016 flood event.

The objective of this study was to integrate SWAT and HEC–RAS models in real-time flood simulation in the VGTB river basin and based on the time series analysis of 20 meteorological stations. For the result of SWAT model calibration, a 10-year period was selected (1995–2004) at the Nong Son hydrological station in the VGTB river basin with R^2^ = 0.93, NSI = 0.92, PBIAS = −0.64; and for validation, a 10 year period was selected (2005–2014) with R^2^ = 0.92, NSI = 0.91, PBIAS = 3.77, as shown in Figure 9 and Figure 10. The result of HEC–RAS model calibration on water level at the Cau Lau hydrological station in the VGTB river basin during October 14–19, 2015 flood event with R^2^ = 0.82, NSI = 0.63, PBIAS = 5.5 and validation on water level at the Cau Lau hydrological station in the VGTB river basin during October 31–November 7, 2016 flood event with R^2^ = 0.85, NSI = 0.66, PBIAS = 6.20, as shown in Figure 11 and Figure 12.

**Figure 13:**
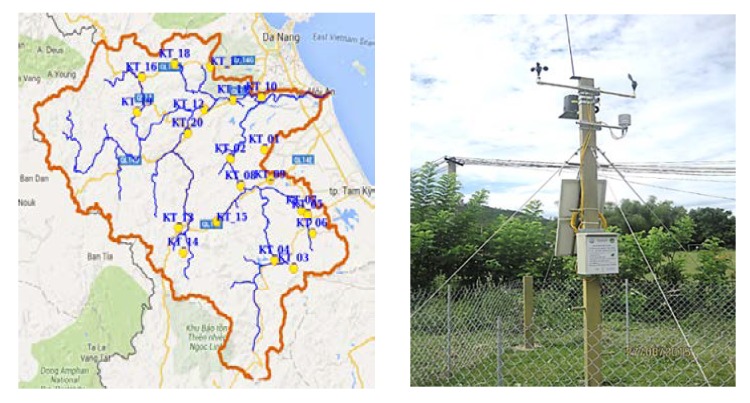
Location (**left**) and sample picture (**right**) of meteorological stations in the Vu Gia-Thu Bon river basin.

**Figure 14:**
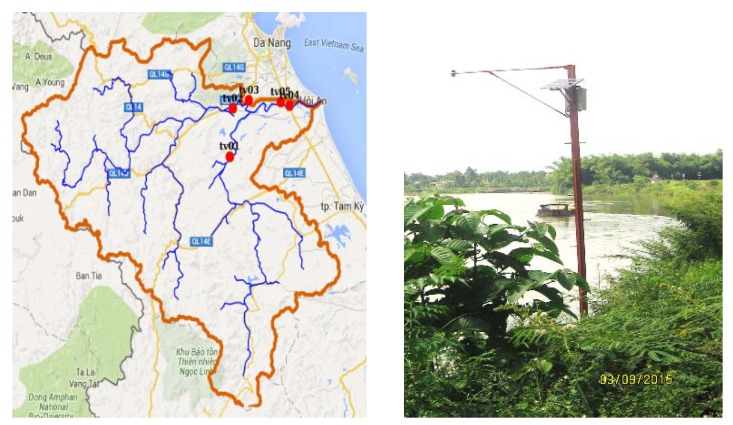
Location (**left**) and sample picture (**right**) of hydrological stations in the Vu Gia-Thu Bon river basin.

**Figure 15:**
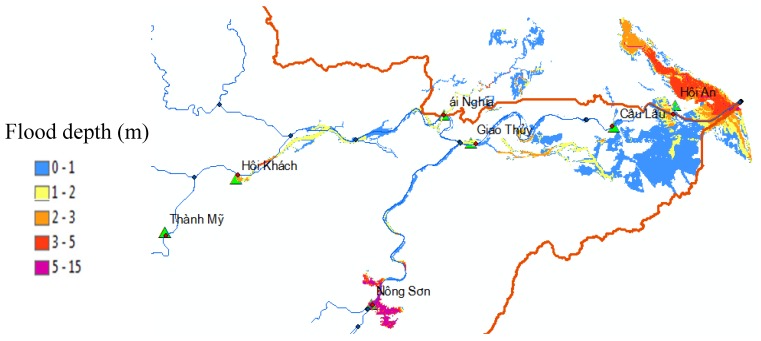
Simulated floodplain map in the lower of the VGTB river basin on November 3, 2016.

Based on the time series analysis of 20 meteorological stations and five hydrological stations in the VGTB river basin as shown in Figure 13 and Figure 14 from August to December 2015, the largest rainfall events occurred on November 3, 2016. The water level predicted by the HEC–RAS model was compared with the precise Digital Terrain Model combined river channels and inundation areas to derive an inundation map showing the spatial extent of the flooding and the water depth at each location. With a processing time of around 5 min, the automated SWAT and HEC–RAS module that simulated flood depth in this event is shown in Figure 15.

### 4.3. Module SMS

Flood data from HEC–RAS is built according to a raster data structure with * .tif file format. Currently, the PostGIS’s spatial data structure integrated into the PostgreSQL database only supports the conversion from raster data in * .tif format to a manual database structure. Because of that, the research team had difficulty in updating automatically raster data into the PostgreSQL database. During the research process, the research team found a solution to overcome this problem. The solution was to convert the raster (* .tif) format to ASCII format (* .txt) with three value fields, XYZ, in which Z is the flood level value with the GDAL command line library (Geospatial Data Abstraction Library). After the data processing starts in the text file, this data will be used and connected to the SMS module to alert the risk of flooding to households in the area and they can be more active in responding to flooding.

**Figure 16:**
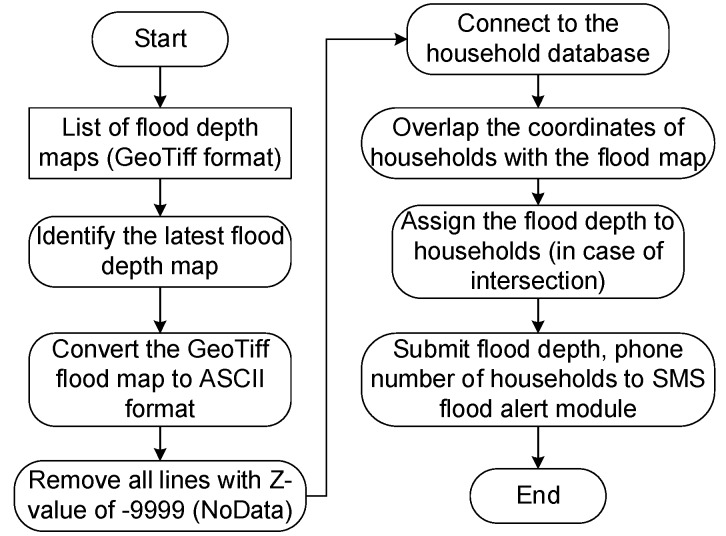
Operation process of household’s flood depth module.

**Figure 17:**
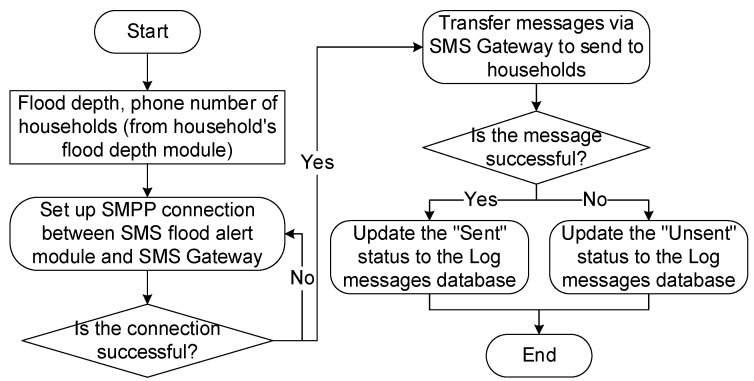
Operation process of short message service (SMS) flood alert module.

The module automatically collects the location of the household (geographical coordinates) with the flood map and sends flood warning information via SMS. The entire operating procedure of the module is illustrated in Figure 16, including the following steps: From the output data of the HEC–RAS model, the flooded area shows the depth of flood in the form * .tif and conducts a selection of flooding data near the most current time. Then, it converts the * .tif file to * .txt format with three X, Y, and Z fields using the GDAL library (where Z is flood depth). It will filter and delete all lines without data (corresponding to Z value of −9999) then reconcile households in the database with flooding data. If it matches, the module will assign the information that shows the depth to each household and save to the value array including flood depth and telephone number of the household. It activates the flood warning module via SMS. This module sends the flood depth information to the phone number and a warning message to each household. The process of activating the flood warning module via SMS is described in Figure 17.

## 5. Conclusions

This paper has presented an automated procedure for forecasting flood events in the VGTB river basin on a sub-daily basis by integrating the SWAT and HEC–RAS models. The whole procedure (from hydro-meteorological data updating, processing to running the SWAT and HEC–RAS models, and visualizing) was packaged into a single, fully automated module that meets strict constraints on accuracy and processing time.

Emergency agencies can make optimal decision-making from the proposed spatial decision support system designed by combining the SWAT and HEC–RAS models. A conceptual framework for implementing the system with interoperability standards has been used. The achieved SDSS results demonstrated the ability to combine real-time meteorological and hydrological models for flood forecasting in the Vu Gia-Thu Bon river basin under a real scenario. Our proposed interoperable standards were appropriate to address the interoperability of heterogeneous data sources, allowing easy addition of new data sources, reuse of data, and access to integrating more data for potential data users. The improvement of the maintainability and assessment of WSN, as well as providing data from poorly gauged or ungauged areas through VGI in the proposed system, can enhance the support of the decision-making.

The SDSS demonstrated accurate prediction of peak flows not only in a long-term assessment (2005–2014) but also in two specific flood events in 2015 and 2016. The accurate prediction of water stages at peak flows and the timing of peaks is key information toward providing early warnings of impending flooding events accurately to the relevant people in the flood zones. These two models are the most popular tools that have been used in a number of watershed studies and river hydraulic analyses. The proposed procedure can be widely adopted and applied to other river basins using both the SWAT and HEC–RAS models.
